# DAGBagM: learning directed acyclic graphs of mixed variables with an application to identify protein biomarkers for treatment response in ovarian cancer

**DOI:** 10.1186/s12859-022-04864-y

**Published:** 2022-08-05

**Authors:** Shrabanti Chowdhury, Ru Wang, Qing Yu, Catherine J. Huntoon, Larry M. Karnitz, Scott H. Kaufmann, Steven P. Gygi, Michael J. Birrer, Amanda G. Paulovich, Jie Peng, Pei Wang

**Affiliations:** 1 Department of Genetics and Genomic Sciences, Icahn School of Medicine at Mount Sinai, New York, NY 10029 USA; 2 Department of Statistics, University of California, Davis, CA 95616 USA; 3 Department of Cell Biology, Harvard Medical School, Boston, MA 02115 USA; 4 Division of Oncology Research and Department of Oncology, Mayo Clinic, Rochester, MN 55905 USA; 5 Division of Oncology Research, Mayo Clinic, Rochester, MN 55905 USA; 6 Winthrop P. Rockefeller Cancer Institute, University of Arkansas for Medical Sciences, Little Rock, AR 72205 USA; 7 Clinical Research Division, Fred Hutchinson Cancer Center, Seattle, WA 98109 USA

**Keywords:** Proteomics, Sensitive and resistant/refractory, Hill climbing, Bootstrap aggregation

## Abstract

**Background:**

Applying directed acyclic graph (DAG) models to proteogenomic data has been shown effective for detecting causal biomarkers of complex diseases. However, there remain unsolved challenges in DAG learning to jointly model binary clinical outcome variables and continuous biomarker measurements.

**Results:**

In this paper, we propose a new tool, DAGBagM, to learn DAGs with both continuous and binary nodes. By using appropriate models, DAGBagM allows for either continuous or binary nodes to be parent or child nodes. It employs a bootstrap aggregating strategy to reduce false positives in edge inference. At the same time, the aggregation procedure provides a flexible framework to robustly incorporate prior information on edges.

**Conclusions:**

Through extensive simulation experiments, we demonstrate that DAGBagM has superior performance compared to alternative strategies for modeling mixed types of nodes. In addition, DAGBagM is computationally more efficient than two competing methods. When applying DAGBagM to proteogenomic datasets from ovarian cancer studies, we identify potential protein biomarkers for platinum refractory/resistant response in ovarian cancer. DAGBagM is made available as a github repository at https://github.com/jie108/dagbagM.

**Supplementary Information:**

The online version contains supplementary material available at 10.1186/s12859-022-04864-y.

## Background

Ovarian cancer is the most lethal gynecological malignancy and is often diagnosed at an advanced stage [1]. Tumor resistance to chemotherapy is a major factor determining the survival of ovarian cancer patients, which has been daunting and remained largely unchanged during the past few decades, despite all the efforts and resources devoted to genomic research of this disease. Therefore, there is an urgent need to develop novel approaches to gain insights of the molecular mechanism for treatment responses to chemo-therapy among ovarian cancer patients.

Recent breakthrough in proteomics research has made it possible to monitor tens of thousands of proteins in one biological sample simultaneously. High-throughput proteomics experiments have been performed on ovarian tumor samples by the Clinical Proteomic Tumor Analysis Consortium (CPTAC) [2, 3], which provides an unprecedented opportunity to screen for potential protein biomarkers that may not otherwise be discovered using previously defined genomic approaches due to the large amount of post-translational modifications in cells. In addition, like most other cancers, ovarian cancer is a complex disease, involving complicated pathway interactions and dysfunctions across multiple biological processes. Thus, a systems-level approach is the key for enhancing our understanding of molecular mechanism underlying the disease, and for detecting effective biomarkers for treatment response. Consequently, higher order molecular networks could serve as central tools for extracting relevant information from high dimensional proteogenomic data. Therefore, in this paper, we aim to screen for proteins having causal associations with response to treatment in ovarian cancer, via constructing appropriate network models based on CPTAC proteogenomics data.

Under the context of protein regulatory networks, edges characterize the dependence relationships among protein abundances in the cell. These relationships can provide insights about steps for the formation of protein complexes or the signaling pathways relating to the drug targets. Specifically, in this paper, we are interested in identifying proteins driving the chemo-response. The resulting candidates could cast light on potential therapeutic targets. Among different approaches to constructing networks, directed acyclic graph (DAG) models, also known as Bayesian network models, are often used to infer causal relationships. A DAG model is a probabilistic graphical model representing a set of variables as nodes and their conditional dependencies as edges via a directed acyclic graph [4–8]. In the past decade, DAG models have been successfully applied to infer causality of the complex regulatory relationships among various entities, such as genes and proteins [9–15], or to identify causal associations between biomarkers and clinical variables [16, 17].

Despite considerable efforts and many pioneering works, there remain challenges in DAG structure learning, especially when the node set contains both continuous and discrete variables (i.e., mixed types of nodes). For example, the clinical outcomes are often binary endpoints, e.g., patient response to treatment, whereas potential biomarkers are often continuous measures such as protein expression levels. While discretization of continuous nodes is a commonly used strategy in DAG learning [18], it does not always guarantee the preservation of the original dependence structure and may also lead to loss of information. On the other hand, simply treating discrete variables as continuous variables leads to model misspecification and false edges/directions. Some works tackle this challenge through imposing constraints on the parent/child status of the binary/continuous nodes [15, 19], which are only suitable for specific types of applications. Two recent works [20, 21] employ hybrid strategies to estimate joint likelihoods of mixed nodes and to infer the edge direction in subsequent steps.

In this paper, aiming for detecting biomarkers causally associated with clinical variables of interest, we propose a score based DAG structure learning algorithm, DAGBagM, which models the continuous nodes by conditional Gaussian distributions and the binary nodes through logistic regressions. Compared to alternative strategies [20, 21], DAGBagM has more flexible distributional assumptions. Moreover, unlike in [15, 19], DAGBagM allows for both continuous and binary variables to be children nodes and is not biased towards a particular type of edges. In addition, since DAGBagM is a purely score based method, it does not involve any tuning parameters and thus is computationally very efficient. To better tackle computational challenges associated with mixed nodes and high dimensionality, we also develop an efficient implementation of the hill climbing algorithm where at each search step information from the previous step is utilized to speed up both score calculation and acyclic status check.

DAG structure learning tends to be highly variable: the learned graph could change drastically with small perturbation of the data. To tackle this challenge, DAGBagM employs an aggregation procedure inspired by bootstrap aggregating (bagging) [22] and couples this procedure with the score-based algorithm. As shown by simulation experiments, this aggregation strategy can greatly reduce false positives with only moderate sacrifice in power.

DAGBagM is also flexible in taking into prior information. This can be important in DAG learning as edge directions are not always identifiable without external information. Independent sources such as time course experiments could provide valuable information on regulatory directions. On the other hand, since prior knowledge/information can be inaccurate, DAGBagM utilizes prior information in an innovative way, i.e., through the aggregation process, to enhance robustness.

In the real data application, we construct DAG models to screen for proteins causally associated with response to treatment in ovarian cancer. We implemented an integrative learning pipeline using DAGBagM to borrow information across multiple proteogenomic data sets of ovarian cancer. The results shed light on the underlying role of the key markers of the metabolic pathways that are causally associated with response to treatment.

## Results

### Simulation experiments

We conduct simulation experiments to examine the proposed DAGBagM algorithm and compare it to several existing DAG structure learning algorithms including PC-alg, and MMHC and the hill climbing (HC) algorithm implemented in the widely used bnlearn package, as well as mDAG [21], a recent method that is specifically designed for learning DAGs with mixed types of nodes.

#### Simulation setup

We perform four sets of simulation experiments. In Simulation (i), we consider the simple case with only continuous nodes. In Simulation (ii), we focus on a low dimension example of mixed nodes (10 continuous and one binary), and evaluate the impact of sample sizes (n = 50, 75, 100) on method performance. In Simulation (iii), for a fixed sample size of n = 100, we consider different dimensions of continuous nodes (p = 20, 60, 120, 200, 500) plus one binary node. In the end, we performed Simulation (iv) to evaluate how the usage of prior edge information may help to improve edge detection, using an example of 200 continuous nodes and 10 binary nodes.

For Simulation (i), given the true graph $G$, $n$ i.i.d. samples are generated according to Gaussian linear mechanisms: ${X}_{i}={\sum }_{j\in p{a}_{i}^{G}}{\beta }_{ij}{X}_{j}+ {\epsilon }_{i}, i=1,\dots ,p$. In this model, ${\epsilon }_{i}$’s are independent Gaussian random variables with mean zero and variance ${\sigma }_{i}^{2}$. The coefficients ${\{\beta }_{ij}\}$ are uniformly generated from $\left[-0.5,-0.3\right]\cup \left[\mathrm{0.3,0.5}\right]$, and the noise variances ${\sigma }_{i}^{2}$s are chosen such that for each node the corresponding signal-to-noise-ratio $\left(SNR\right)$, defined as the ratio between the standard deviation of the signal part and that of the noise part in the linear mechanisms, is within $\left[\mathrm{0.5,1.5}\right]$. For nodes without parents, we simply sample from the standard Normal distribution. For comparison, we consider the non-aggregated hill climbing algorithm (HC) (based on the implementation in DagBagM), the constraint-based algorithm PC-alg (implemented in R package pcalg [23] with $\alpha =0.005$ as suggested in [5]), and the hybrid algorithm MMHC (implemented in R package bnlearn [18] with the default tuning parameter set at $0.05$). Simulation (i) serves the purpose to show the (comparative) performance of DAGBagM when there are only continuous nodes as well as demonstrates the effectiveness of aggregation in reducing false positive edges.

For Simulations (ii), (iii) and (iv), we used a similar data generating scheme for the continuous nodes as in Simulation (i). The binary nodes, denoted by ${Y}_{k}$, are generated by logistic regression models: $P\left({Y}_{k}=1\right)={\gamma }_{k0}+{\sum }_{j\in p{a}_{i}^{G}}{\gamma }_{kj}{X}_{j}$. For all these three simulations, we considered three alternative methods for comparison, namely DAGBagC, mDAG and bnlearnD. In DAGBagC, we simply apply the DAGBagM algorithm while treating all nodes (including the binary nodes) as continuous. For bnlearnD, we first discretize every continuous node using the median cut-off criterion, and we then treat all nodes as binary nodes and apply the “hc” function with BIC score implemented in the R package bnlearn. For mDAG, we use the default parameters setting in the R package mDAG. For a fair comparison, for both bnlearnD and mDAG, we learn one DAG on each bootstrap resample and apply our proposed aggregation algorithm to obtain the final aggregated DAG. Simulations (ii), (iii) and (iv) serve the purpose to show the (comparative) performance of DAGBagM when there are both continuous and binary nodes.

We consider DAGs with different numbers of nodes $\left(p\right)$ as well as different sample sizes $\left(n\right)$. The topology of the true DAGs are shown in the Additional file 1: Figs. S1, S2 and S3. We evaluate the performance of various methods by assessing their powers or true positive rates (TPRs), false discovery rates (FDRs), and the F1 scores for: (i) detection of skeleton edges (i.e., without direction); and (ii) detection of directed edges between continuous and binary nodes. Power (or TPR) is calculated as the ratio between the number of correctly identified edges in the estimated DAG to the number of total edges in the true DAG. FDR is calculated as the ratio between the number of falsely identified edges in the estimated DAG to the number of total edges in the estimated DAG. F1-score is calculated as $F1=\frac{2\times precision \times recall }{(precision+recall)}$, where precision = 1 − FDR and recall = power. For each simulation setting, the performance metrics are averaged across 100 independent replicates.

#### Simulation results

For Simulation (i), we first consider an empty graph ($\parallel E\parallel =0$ edge) to illustrate the effect of aggregation in reducing the number of false positive edge detections. Note that here any detected edge would be a false positive. DAGBagM results in very few false positives, whereas the three non-aggregation methods, namely, HC, PC-alg and MMHC, all have a large number of false positives (Table 1). This experiment demonstrates the effectiveness of aggregation in reducing false positives for DAG learning algorithms.

**Table 1:** Simulation (i)—only continuous nodes

Method	Total # of false edges
DAGBagM	5.7 (2.83)
HC	1995.6 (2.12)
PC-alg	946.5 (12.47)
MMHC	4199.6 (45.7)

True DAG: $p=1000$ nodes, $\parallel E\parallel =0$ edge; sample size $n = 250$. The reported numbers are averaged over 100 replicates and the numbers in parentheses are standard deviations

We then consider a graph (Additional file 1: Fig. S1) with $p=504$ nodes and $\parallel E\parallel =515$ edges under two sample sizes, $n=100$ and $n=250$. It is clear from Additional file 1: Fig. S4 that DAGBagM outperforms the other three methods in terms of balancing power and FDR. It is also obvious that the larger sample size leads to better performance for all methods, especially so for DAGBagM.

For Simulations (ii) and (iii)—mixture of continuous nodes and a single binary node, we consider graphs (Additional file 1: Fig. S2) with different combinations of number of nodes and edges. In each graph, there is a single binary node which is the child of two continuous parents, and it in turn is the parent of one continuous child. These settings mimic the second step in the ovarian cancer application where we learn DAGs on modules containing a few scores of continuous biomarkers and one binary clinical outcome.

**Fig. 1:**
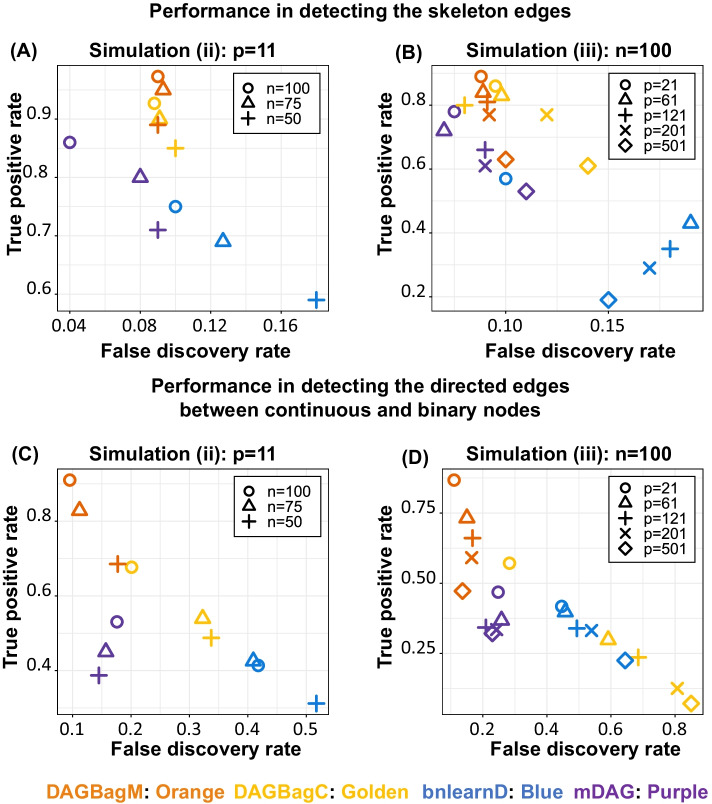
Results for Simulation (ii)—different sample size $n$ with fixed $p=11$ and Simulation (iii)—different number of nodes $p$ with fixed $n=100$. **A**, **B** Performance in detecting the skeleton edges for Simulation (ii) and (iii), respectively. **C**, **D** Performance in detecting the directed edges between continuous and binary nodes for Simulation (ii) and (iii), respectively

We summarize edge detection results in Fig. 1A, C under varying sample sizes for a fixed graph (Additional file 1: Fig. S2A) with $p=11$ nodes and $\parallel E\parallel =8$ edges (Simulation (ii)). Again, the performance of all four methods improves with the increase of sample size $n$ (Fig. 1A). In parallel, we also report edge detection results (Fig. 1B, D) for graphs with different combinations of $p$ and $\parallel E\parallel$ (Additional file 1: Fig. S2B, S2C, S2D and S2E) for a fixed sample size $n=100$ (Simulation (iii)). For both simulations, in terms of power and FDR of skeleton edges detection (Fig. 1A, B), the overall performances of DAGBagM and DAGBagC are quite similar as there is only one binary node, followed by mDAG, while the performance of bnlearnD is much worse. As to the detection of the directed edges between the continuous and binary nodes (Fig. 1C, D), we observe an enhanced performance of DAGBagM over the other three methods for all settings.

**Fig. 2:**
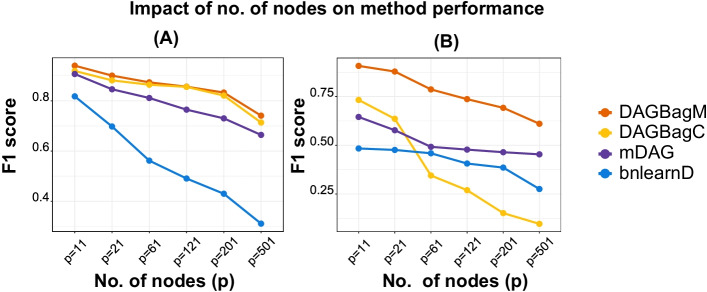
Results for Simulation (iii): impact of graph dimension $\left(p\right)$ on the performance of various methods. **A** F1 scores for detecting the skeleton edges. **B** F1 scores for detecting the directed edges between continuous and binary nodes

Based on the results of Simulation (iii), we further illustrate the impact of graph dimensionality (i.e., the number of nodes $p$) on the performance of DAGBagM and the other methods in terms of the F1 score. The number of nodes has a negative impact on the performances of all methods (Fig. 2). Specifically, the F1 score of DAGBagM for the skeleton edges detection drops from $0.83$ to $0.74$, when the dimension of the graph increases from 201 to 501. We also see that, while all methods demonstrate deterioration in performance with the increase of the graph size, DAGBagM outperforms the other methods for all settings considered.

**Fig. 3:**
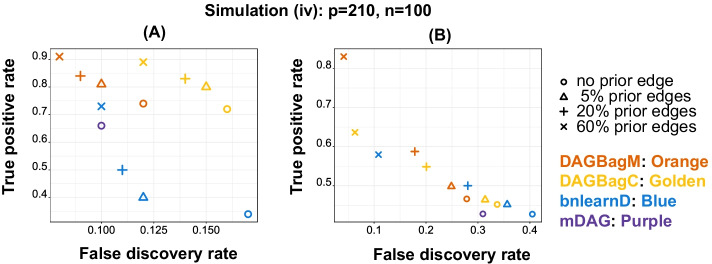
Results for Simulation (iv): impact of prior edges. **A** Performance in detecting the skeleton edges. **B** Performance in detecting the directed edges between continuous and binary nodes. Note that since mDAG does not allow the inclusion of prior edges, we only show its performance for the "no prior edge" setting

For Simulation (iv)—mixture of continuous nodes and multiple binary nodes, we consider a graph (Additional file 1: Fig. S3) with $p=210$ nodes (200 continuous and 10 binary) and $\parallel E\parallel =170$ edges. Furthermore, we randomly sample $5\%,20\%$ and $60\%$ edges from the true DAG and include them as prior edges using the “whitelist” feature in DAGBagM, DAGBagC and bnlearnD. We also considered each method without including any prior edge (“no prior edge”). Since the mDAG package does not provide a way to include prior edges, only results under the “no prior edge” setting are shown for mDAG. We plot power against FDR for detecting the skeleton edges (Fig. 3A) and those for detecting the directed edges between continuous and binary nodes (Fig. 3B). We clearly see an enhanced performance of DAGBagM over the other methods for overall skeleton edges detection as well as directed edges detection between continuous and binary nodes. Moreover, as expected, the performance improves with the inclusion of more true edges as priors.

Furthermore, we check the robustness of DAGBagM with respect to distributional assumptions. We simulate the residual terms of the linear mechanism from non-Gaussian distributions including t-distributions with degrees of freedom 3 or 5, and (mean-centered) Gamma distribution with the shape parameter 1 and the scale parameter 2. In Additional file 1: Fig. S5 we plot the probability density curves of these distributions. We generated the data according to a DAG with the number of nodes $p=102$ and the number of edges $\parallel E\parallel =109$. The signal-to-noise ratio (SNR) is set to be in the range of $\left[\mathrm{0.5,1.5}\right]$, and a fixed sample size of $n=102$ is considered. We report the power (or TPR), FDR and F1 scores in terms of skeleton edges detection by DAGBagM, averaged over 100 simulations (Table 2). From the table, we can see that the performance of DAGBagM is very stable across different distributions, suggesting that DAGBagM is robust with respect to distributional assumptions.

**Table 2:** Robustness of DAGBagM with respect to distributional assumptions

Distribution	Power (TPR)	FDR	F1-score
Gaussian	0.8245	0.087	0.8665
t distribution (df = 3)	0.8375	0.105	0.8653
t distribution (df = 5)	0.8262	0.093	0.8647
Gamma distribution (shape = 1, scale = 2)	0.8182	0.1	0.8572

The data are generated using a DAG with $p = 102$ nodes and $\parallel E\parallel =109$ edges. The range of signal-to-noise ratio (SNR) is set to be [0.5, 1.5], and the sample size is set to be $n = 102$. The reported numbers are averaged over 100 independent replicates

**Fig. 4:**
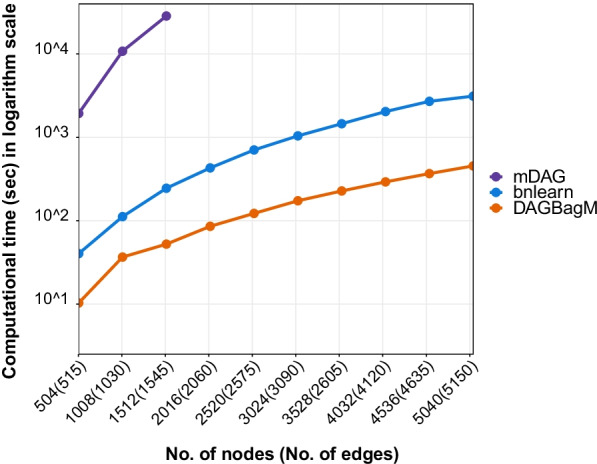
Run time comparison: the Hill Climbing (HC) algorithm implemented in DAGBagM (orange) versus HC implemented in bnlearn (blue) versus mDAG (purple) across a collection of settings with different combinations of number of nodes and number of edges. Note that mDAG was only run for up to $1512$ nodes, as beyond that the computational time increases drastically

In the end, we compare the run time of the HC algorithm implemented in DAGBagM with the HC algorithm implementation in R package bnlearn, as well as with mDAG (using default parameters). We plot the run time as a function of the number of nodes (edges) when the sample size is fixed at $n=500$ and the maximum number of search steps is capped at 1000 (Fig. 4). The run time of mDAG increases dramatically as the number of nodes increases (and therefore only results up to p = 1512 nodes are reported for mDAG), followed by the run time of bnlearn, while the run time of DAGBagM increases at a much slower rate. As an example, when $p=1008, \parallel E\parallel =1030$, it takes $36.68$ s by DAGBagM, $112.05$ s by bnlearn and $10803.2$ s by mDAG to fit the DAG model, on a machine with 8 GB RAM and dual-core CPU.

In summary, the simulation results suggest that aggregation is an effective way to reduce false positives in DAG structure learning, treating continuous nodes and binary nodes using different models are beneficial in presence of mixture types of nodes in terms of both edge and edge direction detection, and inclusion of prior information further improves the performance. In addition, DAGBagM is robust to distributional assumptions, and it is very competitive in terms of computational cost.

### Application to ovarian cancer

High grade serous ovarian cancer (HGSOC) is the most lethal gynecological malignancy, and its daunting overall survival has not changed significantly for decades. Chemotherapy is the main treatment strategy for these patients, and chemo-resistance is the most important factor determining the survival outcomes of HGSOC patients. There is a pressing need to gain a better understanding of resistance mechanisms of chemotherapies. Comprehensive mass-spectrometry based proteomics characterization has been carried out in multiple recent cancer studies. Pathway activities characterized by proteomics data revealed surprising new information of tumor samples. Specifically, metabolic pathways such as Oxidative Phosphorylation and Adipogenesis are the ones showing the least correlation between RNA and proteomics data in multiple tumors, suggesting active post-translational modifications to the members of these pathways in tumors [3]. Metabolic reprogramming, recognized as one of the cancer hallmarks [24], promotes the activation of oncogenes and thus facilitates cancer progression and metastasis [25]. This motivates us to screen for potential protein markers in related pathways in ovarian cancer based on newly generated proteomics data, which might lead to new insights missed in previous genomic based studies. Specifically, we apply DAGBagM on ovarian cancer proteogenomic data sets, focusing on Oxidative Phosphorylation and Adipogenesis pathways, to derive a causal graph to characterize the dependence of patient's response to chemotherapy on protein marker activities.

**Fig. 5:**
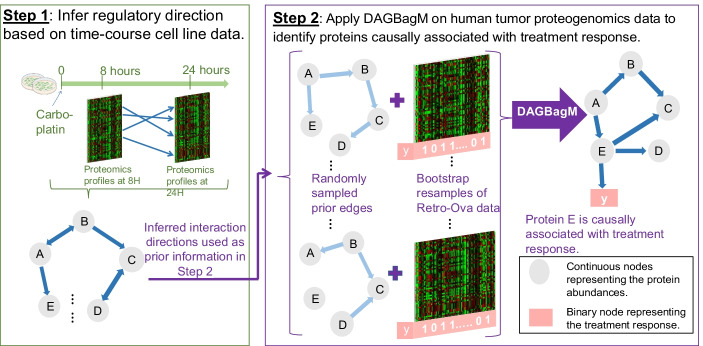
Ovarian cancer application: integrative DAG learning pipeline. Step 1: infer a directed network based on the time course cell line proteogenomics data from a treatment perturbation experiment. Note that, there could be bidirectional edges when we drop the time suffix in the resulting network from this step. Step 2: infer outcome-driven-DAGs with both continuous (e.g. proteins) and a binary variable (treatment response) based on CPTAC ovarian cancer proteogenomics data, while taking into account the directional information learned in Step 1

The detailed data analysis pipeline is described in Fig. 5 and Additional file 2: Method S3. Briefly, the pipeline consists of two major steps. We first derive prior information on causal protein–protein interactions using a time-course ovarian cancer cell line proteogenomic data set from a treatment perturbation experiment (Step 1) [26]. Then, using the direction information learned from Step 1 as priors, we construct an outcome-driven-DAG for genes in the Oxidative Phosphorylation and Adipogenesis pathways using a tumor proteogenomic data set from the CPTAC retrospective ovarian (Retro-Ova) cancer study (Step 2) [3]. Throughout, we focus on the 260 genes from the Oxidative Phosphorylation and Adipogenesis pathways that were measured in both proteogenomics data sets.

#### Step 1: Learning regulatory direction on time-course cell line data

We first use a time-course cell line proteomics data [26] to generate initial information on regulatory edge directions among the 260 proteins. The data contains proteomic profiles of 6 ovarian cancer cell lines, with three biological replicates of each cell line, from two different time points, namely, 8-h and 24-h, after a chemo-drug perturbation. Thus, there are 36 proteomic profiles in total. It is reasonable to assume that the activities at the earlier time point drive the protein abundances at the later time point. Thus, for each protein, we treat its measurements at the two time points as two separate nodes and we exclude edges from nodes at the 24-h time point to nodes at the earlier 8-h time point. By applying the DAGBagM algorithm, we identify 100 directed edges among the 520 nodes. Out of these, 81 nodes have at least one child and 100 nodes have at least one parent. Due to the small sample size, we can expect that the estimated DAG contains false edges and only a subset of the regulatory relationships are identified. However, this provides valuable prior information for the Step 2 analysis based on a larger ovarian tumor data set.

Note that there could be bidirectional edges in the above network, when the time information is dropped. For example, there might be an edge from the 8-h time point of protein A to the 24-h time point of protein B (A_8_ → B_24_) as well as another edge from protein B at 8-h time point to protein A at 24-h time-point (B_8_ → A_24_). These are illustrated as A ↔ B in Fig. 5 under Step 1. Thus, in Step 2, when sampling a subset of edges from the inferred cell-line network to form the prior “whitelist”, we include further steps to make sure that the sampled edge set does not violate acyclicity (Additional File 2: Method S3).

#### Step 2: Identifying protein biomarkers causally associated with treatment response using the Retro-Ova data

In this step, we seek for potential protein markers causally associated with chemotherapy resistance based on the Retro-Ova proteomics data, which contains proteomic profiles of treatment naive primary tumor samples of 174 ovarian cancer patients [3]. We first use the overall survival (OS) information of these patients to derive an approximation of patient response to treatment: patients with $OS>5.5$ years were labeled as sensitive, and patients with extremely short survival time $(OS<1.5$ years) were labeled as resistant/refractory. There are 43 patients classified to the sensitive group, and 36 to the resistant/refractory group, so a total of 79 patients are used for the subsequent DAG inference. Data preprocessing is described in the Additional file 2: Method S4.

Note that since overall survival (OS) is a complicated phenotype, we want to focus on cases with unambiguous treatment responses in order to have strong signals in the DAGBagM analysis. According to [27], the definition of resistant/refractory for ovarian cancer patients is as follows: platinum-resistance describes disease that recurs within 6 months (0.5 year) of last therapy; and refractory disease is a form of platinum-resistance where the cancer progresses during treatment or recurs within 1 month after platinum chemotherapy completion. Assuming that the treatment spans about 6 months (0.5 year) after diagnosis and the patients tend to have a short life span after tumor relapse, we use 1.5 year of OS cutoff (*i.e.* 0.5 year for the treatment period, plus 0.5 year from treatment to tumor relapse for resistant definition, and plus 0.5 year from tumor relapse to death). This stringent cutoff is meant to avoid including (borderline) sensitive cases in the resistant/refractory group. For example, a sensitive patient who relapses 7 months after treatment could have an overall survival time shorter than 2 years and thus would be labeled as resistant if a less stringent (e.g. 2-year or more) OS cutoff had been adopted. On the other hand, we used cutoff 5.5 (0.5 year for treatment period plus additional 5 years of survival) to select patients who clearly had excellent responses to treatments and did not relapse in the initial few years.

**Fig. 6:**
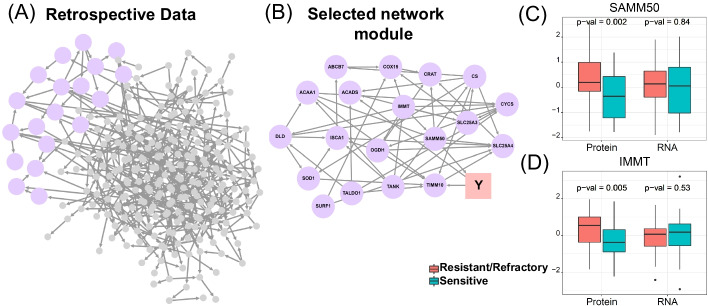
Ovarian cancer application. **A** DAG learned from the Retro-ova data. There are 260 nodes (proteins) and 464 inferred edges. The purple module is of particular interest, as it contains a parent node SAMM50 of the treatment response. **B** Topology and node information for the purple module in **A**. **C**, **D** Boxplots of protein abundance and RNA expression level of SAMM50 and IMMT in the sensitive and resistant/refractory tumors based on Retro-Ova data. The *p* values are calculated using Student’s t-test

To enhance power, we further divide the 260 proteins into a few smaller tightly correlated functional subgroups and infer outcome-driven-DAG for each subgroup separately. Specifically, we first learn a DAG for the 260 proteins (without the treatment response variable) based on the Retro-Ova data using the selected 79 patients. We then identify well-connected network modules in this DAG to define protein functional groups (Fig. 6A). This reveals several well-connected network modules, each containing roughly 10–20 proteins after we apply the "cluster_edge_betweenness" function implemented in the R package igraph [28]. The detail is given in the Additional file 2: Method S3.

We then infer outcome-driven-DAGs for each of these modules based on the Retro-Ova data of the 79 labeled patients. For each module, we apply DAGBagM to the abundance measures of proteins in the module together with a binary variable $Y$ representing the response to treatment status (sensitive vs. refractory). We also incorporate prior information for edge directions from Step 1 by specifying whitelists (i.e., edges always included) when fitting DAGBagM. Note that, since some of the directions learned from the cell-line data could be either false positives or do not apply to tumor cells, along with each bootstrap resample of the data, we randomly sample only a subset of the inferred edge directions from Step 1 to form a whitelist. By this way, false edges from Step 1 would only impact a subset of the DAGs in the learned ensemble and thus are more likely to be filtered out during the aggregation step.

The resulting outcome-driven-DAGs suggest a few causal protein markers for treatment response. Specifically, DAGBagM identified 11 genes as the parent nodes of the response based on the proteomics data (Table 3). The estimated DAG of the purple-colored module (Fig. 6B) is of particular interest, in which SAMM50 is a parent node causally associated with the response to treatment, with an upstream regulator, IMMT. For both SAMM50 and IMMT, significantly higher protein abundances are observed in resistant/refractory tumors than in sensitive tumors (Fig. 6C, D, *p* values < 0.05 based on Student’s t-tests). Protein of SAMM50 is a component of the sorting and assembly machinery of the mitochondrial outer membrane. It was hypothesized that change in the transport of proteins and metabolites into mitochondria due to SAMM50 is responsible for the energy production in cancer cells, and thus activity of SAMM50 has been suggested to be predictive of cancer progressing in breast cancer [29]. SAMM50 protein is closely associated with the mitochondrial contact site and cristae organizing system (MICOS) complex [30], of which IMMT is an important player. Recently, a study has revealed the prognostic value of IMMT protein in gastric cancer [31]. Thus, we further evaluate the prognostic value of SAMM50 and IMMT protein in the complete Retro-Ova cohort (n = 174) using Cox regression models. The protein abundances of both SAMM50 (Cox regression *p* value = 0.0381, hazard ratio = 5.104) and IMMT (Cox regression *p* value = 0.0376, hazard ratio = 3.535) are significantly associated with poor survival (Additional file 1: Fig. S10A–B). Furthermore, we observe that abundances of both SAMM50 and IMMT are associated with 3-year survival outcomes (Student’s t-test *p* values < 0.05, Additional file 1: Fig. S10C–D) but not associated with 5- or 10-year survival outcomes. This might be due to the fact that 3-year survival outcome is greatly driven by the initial treatment responses, while longer year survival outcomes depend on many other factors. These results, for the first time, suggest the potential roles of proteins of SAMM50 and IMMT in platinum treatment response among ovarian cancer patients.

**Table 3:** Genes/proteins identified as parents of the treatment response by DAGBagM, bnlearnD and mDAG based on Retro-ova protein and RNA data

Method	Protein	RNA
DAGBagM	SAMM50*, NDUFS4, NQO2, ME1, UQCRC1, AIFM1, NDUFAB1, HSPA9, RETSAT, NDUFB4, CYB5A	SLC25A3, CPT1A, NMT1, AGPAT3, TIMM50*
bnlearnD	MGST3, PEX14, FAH, SUCLA2	HTRA2, STAT5A*, SLC25A3
mDAG	FAH, TIMM50*	STAT5A*, COX6B1, NDUFB7

Genes with * indicates their association with overall survival (Cox regression *p* value < 0.05) using the complete (n = 174) Retro-Ova data

In addition, we apply bnlearnD and mDAG on the Retro-Ova proteomics data in the same manner. Both methods detect fewer parent nodes of treatment response than DAGBagM (Table 3). Specifically, for the purple module (Fig. 6B), bnlearnD identifies no parent node while one child node of the treatment response (Additional file 1: Fig. S7). And mDAG does not identify any protein as either parent or child node for the treatment response for this module (Additional file 1: Fig. S8).

Moreover, to compare DAGs derived based on protein abundances to that of RNA expression levels, we apply DAGBagM, bnlearnD, and mDAG to the Retro-ova RNAseq data with prior edge information learned from the cellline RNAseq data using the same procedure. DAGBagM, bnlearnD and mDAG inferred 5, 3, and 3 genes as the parent nodes of the response, respectively (Table 3). Interestingly, these are mostly non-overlapping with those identified based on the proteomics data (Table 3). Specifically, neither SAMM50 nor IMMT has been inferred as a parent node based on the RNAseq data. This is further illustrated in Fig. 6C, D: no significant association is detected between RNA expression levels of either SAMM50 or IMMT with response to treatment (Student’s t-test *p* values > 0.05). Since proteins of SAMM50 and IMMT relate to the MICOS protein complex, it is likely that their protein abundances were greatly influenced by post-translational modifications during complex forming and/or activation. Indeed, RNA expression level and protein abundance of both SAMM50 and IMMT in the same tumor samples showed poor correlation (cor < 0.25, Additional file 1: Fig. S6).

In summary, DAGBagM identified more parent nodes as compared to bnlearnD or mDAG, which is consistent with the observation that it has higher power in edge detection than other methods in the simulation studies. Moreover, our findings nicely illustrate that the proteomics data and RNA-seq data provide complementary information on searching for biomarkers associated with treatment response.

## Discussion

In this paper, we propose DAGBagM, a novel DAG structure learning tool for data with both continuous and binary variables using a score-based method coupled with bootstrap aggregation. The score-based DAG structure learning algorithm allows either type of nodes to be a child node. As shown by simulation experiments, DAGBagM achieves better performance for detecting edges and edge directions, compared to conventional strategies that treat all nodes as one type. It also outperforms a recent DAG method—mDAG—that is designed for mixed types of nodes. The flexibility and competitive performance provided by DAGBagM have important relevance in practice when one is interested in detecting biomarkers causally associated with clinical outcomes, as the latter are often binary endpoints and the former are often continuous measurements. In addition, DAGBagM employs a novel technique to aggregate DAGs learned on bootstrap resamples, which can greatly reduce the number of false positives. Moreover, this aggregation procedure is a general tool that can be coupled with any structure learning algorithm (score based or not) and is a flexible and robust way to incorporate prior information. Finally, our implementation of the hill-climbing algorithm is much faster than that in a widely used DAG learning R package bnlearn as well as mDAG.

We apply DAGBagM to analyze ovarian cancer proteogenomics datasets with the goal to identify potential prognostic proteins in ovarian cancer. To facilitate the inference of edge directions, we utilize a time-course cell line proteogenomics data to get the initial regulatory direction estimation. We then use learned edges as prior information to construct DAGs from another ovarian tumor proteogenomics data set. Our result reveals multiple candidate protein biomarkers, including SAMM50 and IMMT, to be causally associated with response to treatment in ovarian cancer. Proteins of SAMM50 and IMMT are an important regulator and a member of the MICOS complex, respectively. While prognostic values of SAMM50 and IMMT in other cancer types have been reported, our analysis for the first time suggests their prognostic roles in ovarian cancer. Intriguingly, the prognostic value of SAMM50 and IMMT could not be observed based on RNA expression data from the same set of patients, suggesting the importance and great potential of employing proteogenomic integrative analysis in biomedical research. These biomarkers identified to have a causal relationship with response to treatment in ovarian cancer could serve as potential targets to individualized anti-cancer agents, upon evaluation through clinical practice [32]. In this paper, we focus on the Oxidative Phosphorylation and Adipogenesis pathways to investigate the underlying molecular mechanism of their members. Although extending the analysis pipeline developed here to other pathways in a systematic manner is out of the scope of this paper, we see great potential of DAGBagM in systems biology applications. As many other models for constructing high-dimensional networks, DAGBagM is designed to infer sparse networks [5, 33, 34]. In practice, this model works well when the number of edges is in the same order as the number of nodes. This is a reasonable assumption for gene/protein regulatory networks, as each gene/protein is expected to interact with only a limited number of other genes/proteins [35, 36]. Nevertheless, graph dimensionality has a negative impact on the performance of DAGBagM (and other DAG learning methods), as illustrated in Fig. 2. However, as demonstrated in the simulation results, when the number of nodes is around 100, DAGBagM has > 80% power to detect skeleton edges with a sample size around 100. Thus, in real data analysis, when DAGBagM is applied to individual pathways or gene sets of size ~ 100, we expect its power for edge detection to be reasonable.

The current DAGBagM implementation is aimed to achieve fast speed rather than efficient memory usage (memory use is in the order of ${p}^{2}$). DAGBagM runs smoothly for $p$ in the order of ${10}^{3}$ on a machine with 8 GB RAM and dual-core CPU. It can fit larger models (e.g., $p$ in the order of ${10}^{4}$) on small-scale multi-core servers. But for very large models (e.g., $p$ in the order of ${10}^{6}$), memory could become a limiting factor.

In the end, as presented in the simulation, DagBagM is quite robust to distributional assumptions. So, we expect DAGBagM to have robust performance on bulk tissue-omics applications as we demonstrated in the ovarian cancer study using both protein and RNA data. However, DAGBagM may not be appropriate for low-depth read count data with extensive drop-out rates, such as those from single-cell or spatial transcriptomic profiling experiments. Extension of DAGBagM to those applications is warranted as future research.

## Methods

In this section, we present a new tool—DAGBagM—for learning directed acyclic graphs with both continuous and binary nodes.

We first introduce some notations. A directed acyclic graph $G\left(V,E\right)$ consists of a node set $V$ and an edge set $E$ with directed edges from parents nodes to children nodes. In a DAG model, the node set corresponds to a set of random variables and the edge set encodes the conditional dependence relationships among these random variables. DAG structure learning amounts to identifying the parent set (also referred to as neighborhood) of each node in the graph. Although different DAGs could encode the same set of conditional dependencies (which form an equivalent class of DAGs), it is shown that, two DAGs are equivalent if and only if they have the same set of skeleton edges and $v$-structures [8]. The skeleton edges are obtained by removing directions from the directed edges and a $v$-structure is a triplet of nodes $\left({x}_{1},{x}_{2},{x}_{3}\right)$, such that ${x}_{1}\to {x}_{3}\in E,{x}_{2}\to$ ${x}_{3}\in E$, and ${x}_{1},{x}_{2}$ are not adjacent.

To deal with both continuous and binary nodes, DAGBagM models continuous nodes as Normally distributed given their parent nodes; and models binary nodes through logistic regressions with parent nodes as regressors. It then uses an efficient implementation of a popular search algorithm, the hill climbing (HC) algorithm [37], to look for a DAG model that optimizes a score function. Our implementation uses information from the previous search step to facilitate the score calculation and acyclic status check in the current step of the search algorithm. Details are given in two propositions in the Additional file 2: Method S1. DAGBagM also employs a novel aggregation procedure to learn stable structures and to reduce false positive edges. Moreover, DAGBagM can incorporate prior information through blacklist(s) of forbidden edges and/or whitelist(s) of edges that are always kept in the graph. This is done through the exclusion of certain operations from the set of eligible operations at each search step. These lists may be utilized in either the individual DAG learning step or in the aggregation step. Major steps of DAGBagM are summarized in Algorithm 1 and in Additional file 1: Fig. S9. More detailed information is provided in the subsequent subsections. Note that, although we describe the DAGBagM algorithm under the situation when there are both continuous and binary nodes for the most generality, it is applicable when there are only continuous nodes or when there are only binary nodes.

### Score calculation

Structure learning based on likelihood score overfits the data since it always favors larger models, i.e., distributions with less independence constraints/DAGs with more edges. Therefore, it is reasonable to consider scores that penalize for model complexity. Since Bayesian information criterion (BIC) [38] is model selection consistent and locally consistent [39], DAGBagM adopts BIC-type scores to be minimized by the hill-climbing algorithm.

Specifically, for a continuous node, denoted by $X$, at each search step, its score is calculated by regressing $X$ onto its current parent set. Specifically, for a given graph $G$, $s{core}_{BI{C}_{X}}:=\left(\frac{RS{S}_{X}}{n}\right) +\left|p{a}_{X}^{G}\right|log\left(n\right)$, where, $RS{S}_{X}$ is the residual sum of squares, $n$ is the sample size, $p{a}_{X}^{G}$ denotes the parent set of $X$ in graph $G$ and $\left|p{a}_{X}^{G}\right|$ denotes the size of the parent set. For a binary node $Y$, the score is obtained by regressing $Y$ onto its current parent set through logistic regression:

$$\begin{aligned} score_{{BIC_{Y} }} & : = - 2\left( {\sum\limits_{k = 1}^{n} {I\left( {Y_{k} = 1} \right) log \left( {\hat{p}_{k} } \right)} + \sum\limits_{k = 1}^{n} {I\left( {Y_{k} = 0} \right)log \left( {1 - \hat{p}_{k} } \right)} } \right) \\ & \quad + \left| {pa_{Y}^{G} } \right|log(n), \\ \end{aligned}$$

where $Y_{k} \textrm{\ denotes\ the } \ k^{th} \textrm{\ sample \ of} \ Y, \ {\widehat{p}}_{k}=P\left({Y}_{k}=1\mid p{a}_{Y,k}^{G}\right)=\frac{exp \left({\widehat{\gamma }}_{0}+{\widehat{\gamma }}^{T}p{a}_{Y,k}^{G}\right) }{1+exp ({\widehat{\gamma }}_{0}+{\widehat{\gamma }}^{T}p{a}_{Y,k}^{G}) },{\widehat{\gamma }}_{0}$ is the fitted intercept and $\widehat{\gamma }$ is the vector of the fitted coefficients in logistic regression. Finally, the score of a graph $G$ is the summation of individual node’s score: ${score}_{BIC}\left(G:D\right) := {\sum }_{X:Continuous Nodes}{score}_{{BIC}_{X}}+ {\sum }_{Y:Binary Nodes}{score}_{{BIC}_{Y}}$, where $D$ denotes the data.


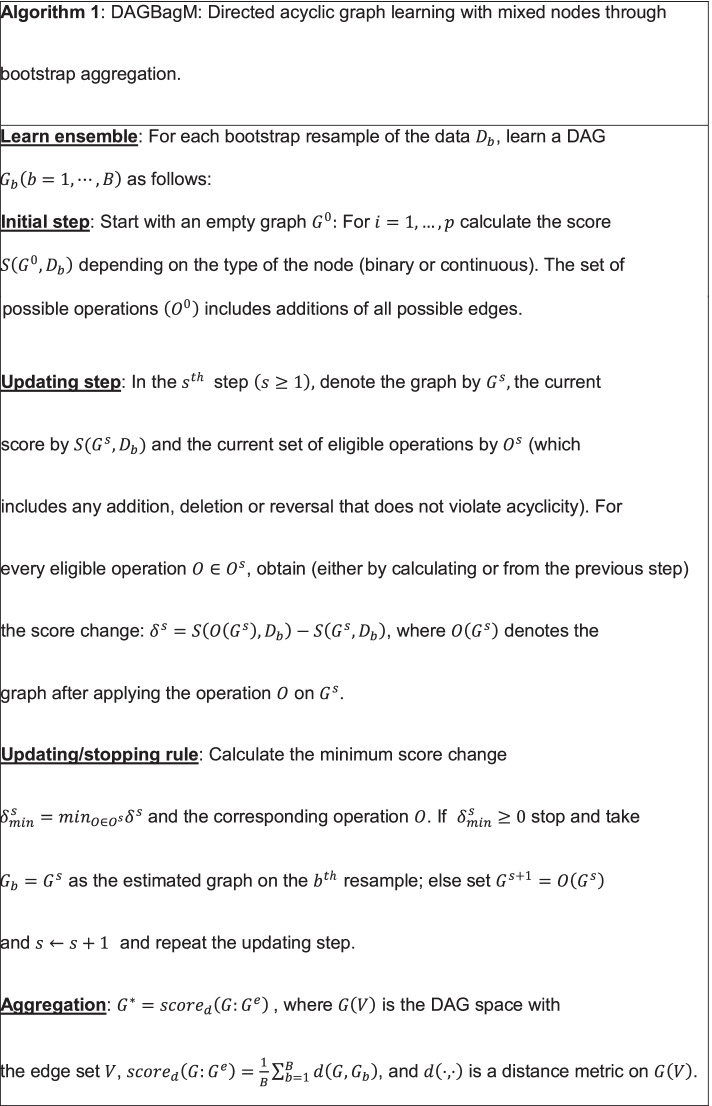


### Bootstrap aggregation

DAGBagM employs an aggregation procedure to learn stable structures and to reduce false positive edges. It first learns an ensemble of DAGs where each DAG is learned on a bootstrap resample of the data. It then obtains an aggregated DAG through minimizing the average structural Hamming distance to DAGs in the ensemble [7, 40].

The aggregation of an ensemble of DAGs is nontrivial because the notion of mean is not straightforward on the DAG space. Here, we generalize the idea of mean by searching for a DAG that minimizes an average distance to the DAGs in the ensemble. For this purpose, we define a distance metric based on the Hamming distance. In information theory, the Hamming distance between two $0{-}1$ vectors of equal length is the minimum number of substitutions needed to convert one vector to another. This can be generalized to give a distance measure between two DAGs with the same set of nodes, defined as the minimum number of basic operations, namely, addition, deletion and (possibly) reversal that are needed to convert one graph to another. This definition leads to a valid distance metric and is referred to as the structural Hamming distance (SHD).

While there are different variants of SHD depending on how the reversal operations are counted, here we focus on the case where the reversal operation is counted as one unit of operation. This leads to the following distance: $d\left(G,\widetilde{G}\right)={\sum }_{1\le i<j\le p}max\left\{\left|A\left(i,j\right)-\widetilde{A}\left(i,j\right)\right|,\left|A\left(j,i\right)-\widetilde{A}\left(j,i\right)\right|\right\}$, where $A$ and $\widetilde{A}$ denote the adjacency matrices of the DAGs $G$ and $\widetilde{G}$, respectively. The adjacency matrix of a DAG is a $0{-}1$ element matrix where the $\left(i,j\right)$-th element is one if there is a directed edge from the $i$th node to the $j$th node; otherwise, it is zero.

Finally, the aggregation score between a DAG $G$ and an ensemble of DAGs ${G}^{e}=\left\{{G}_{1},\cdots ,{G}_{B}\right\}$ is the average distance between $G$ and the DAGs in the ensemble: $scor{e}_{d}\left(G:{G}^{e}\right)=\frac{1}{B}{\sum }_{b=1}^{B}d\left(G,{G}_{b}\right)$. By Proposition 3 in Additional file 2: Method S2, the aggregation score can be expressed as $scor{e}_{d}\left(G:{G}^{e}\right)={\sum }_{e\in E\left(G\right)}\left(1- 2g{p}_{e}\right)+C$, where $C$ is a constant, and $g{p}_{e}$ is a generalized selection frequency. Given an ensemble, one can search for the DAG that minimizes the aggregation score while maintaining acyclicity by applying the HC algorithm. We defer details to the Additional file 2: Method S2.

## Supplementary Information

**Additional file 1:** Supplementary figures.

**Additional file 2:** Supplementary methods.

## Data Availability

We used the cell line data (that was used in Step 1 of our integrative data analysis) from ProteomeXchange Consortium (http://proteomecentral.proteomexchange.org). The dataset identifier is PXD020764. We used the CPTAC retrospective data (referred to as the Retro-Ova data in the manuscript that was used in Step 2 of our integrative data analysis) from Proteomic Data Commons (PDC) (https://pdc.cancer.gov/pdc/). The PDC IDs of the Retro-Ova data are PDC000114 and PDC000113.
