# Supplementary material for: DAGBagM: learning directed acyclic graphs of mixed variables with an application to identify protein biomarkers for treatment response in ovarian cancer
Source: BMC Bioinformatics. 2022 Aug 5;23:321. doi: 10.1186/s12859-022-04864-y (PMC9354326; doi:10.1186/s12859-022-04864-y)
Supplement: Supplementary file 1 — Additional file 1: Supplementary figures. [file 12859_2022_4864_MOESM1_ESM.docx]

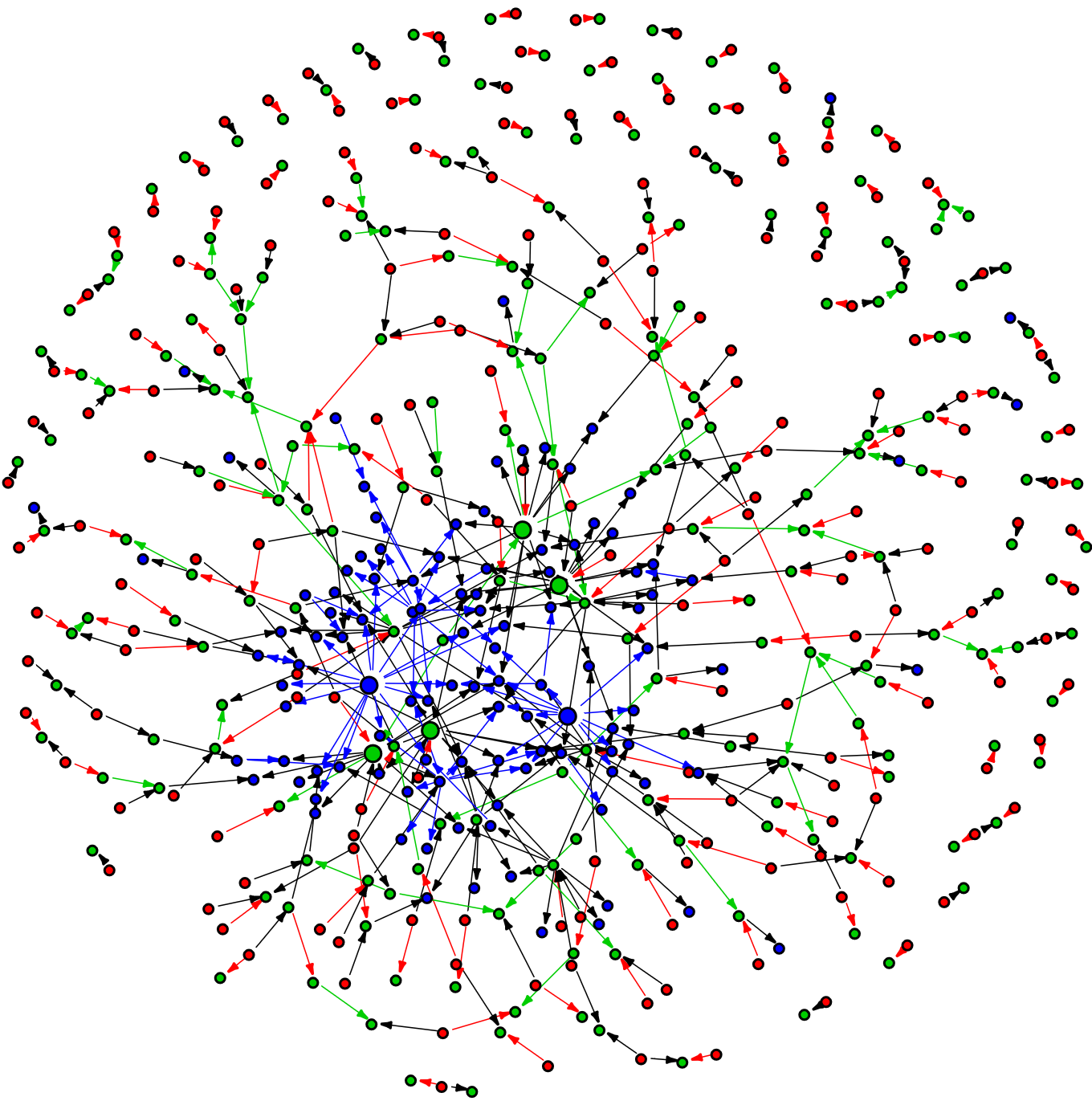


Fig. S1. Graph with $p= 504,\parallel E\parallel= 515$.


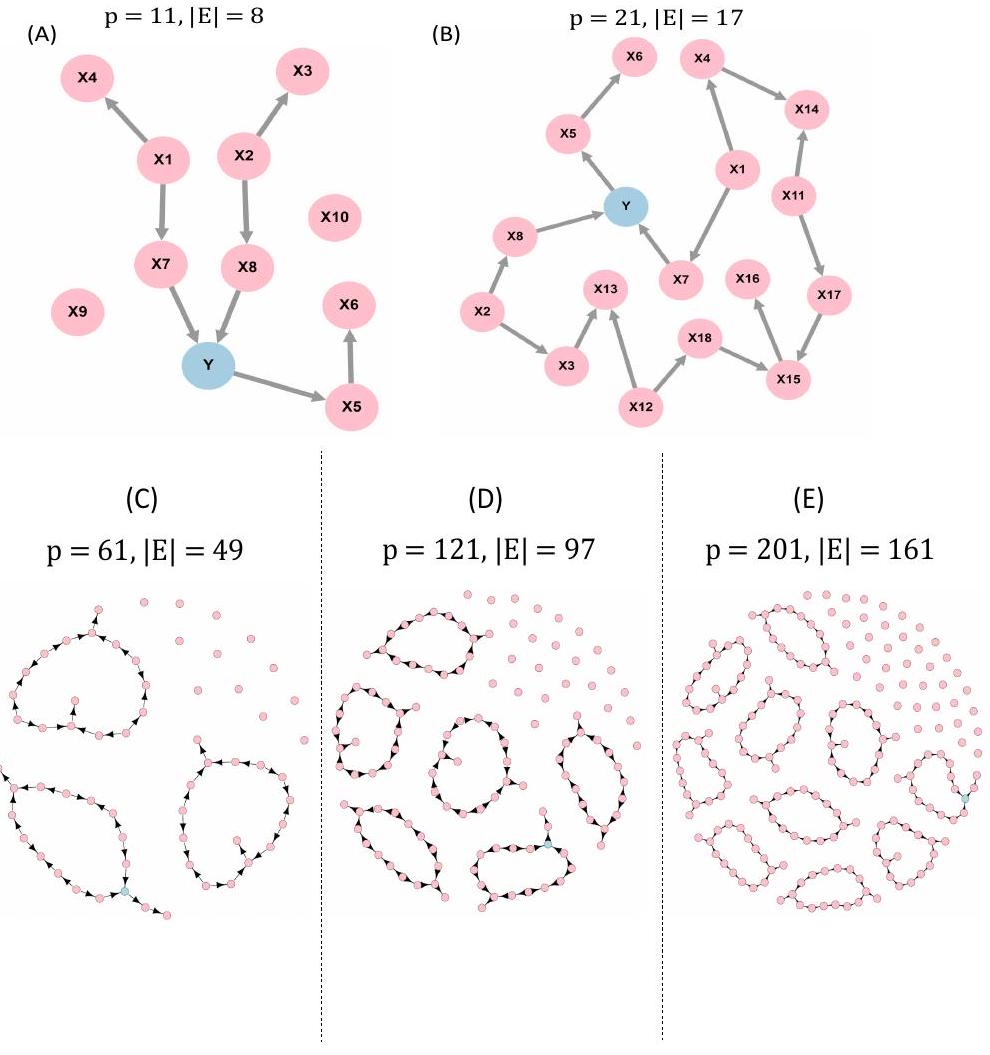


Fig. S2. (A) True DAG with $p= 1$1(10 continuous nodes and 1 binary node), $\parallel E\parallel= 8$, (B) True DAG with $p= 21$ (20 continuous nodes and 1 binary node Y), $\parallel E\parallel=$ $17$, (C) True DAG with $p= 61$ (60 continuous nodes and 1 binary node Y), $\parallel E\parallel= 49$, (D) True DAG with $p= 121$ (120 continuous nodes and 1 binary node Y), $\parallel E\parallel= 97$, (E) True DAG with $p= 201$ (200 continuous nodes and 1 binary node Y), $\parallel E\parallel= 161$.


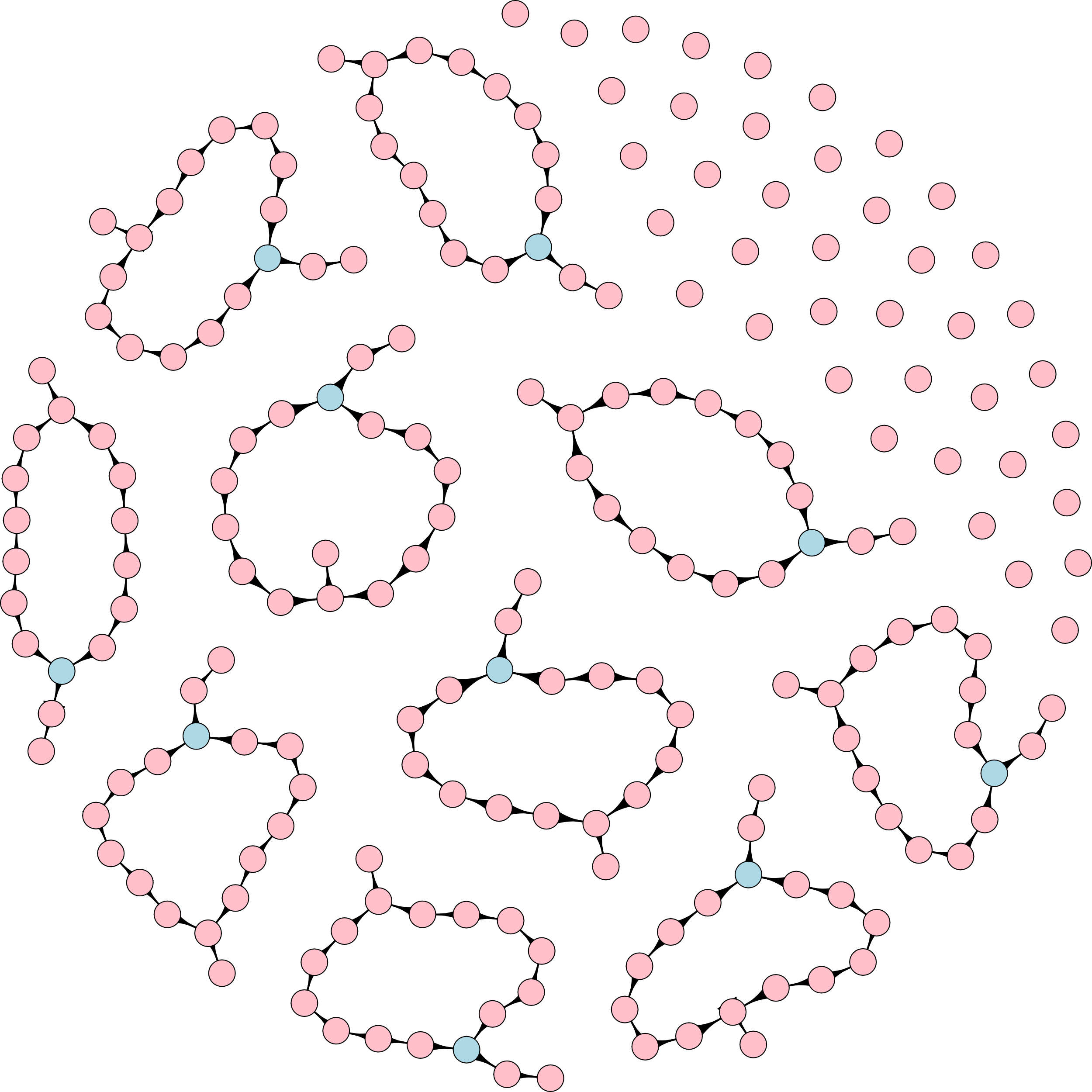


Fig. S3. True DAG with $p= 210$ (200 continuous nodes and 10 binary nodes), $\parallel E\parallel= 170$.


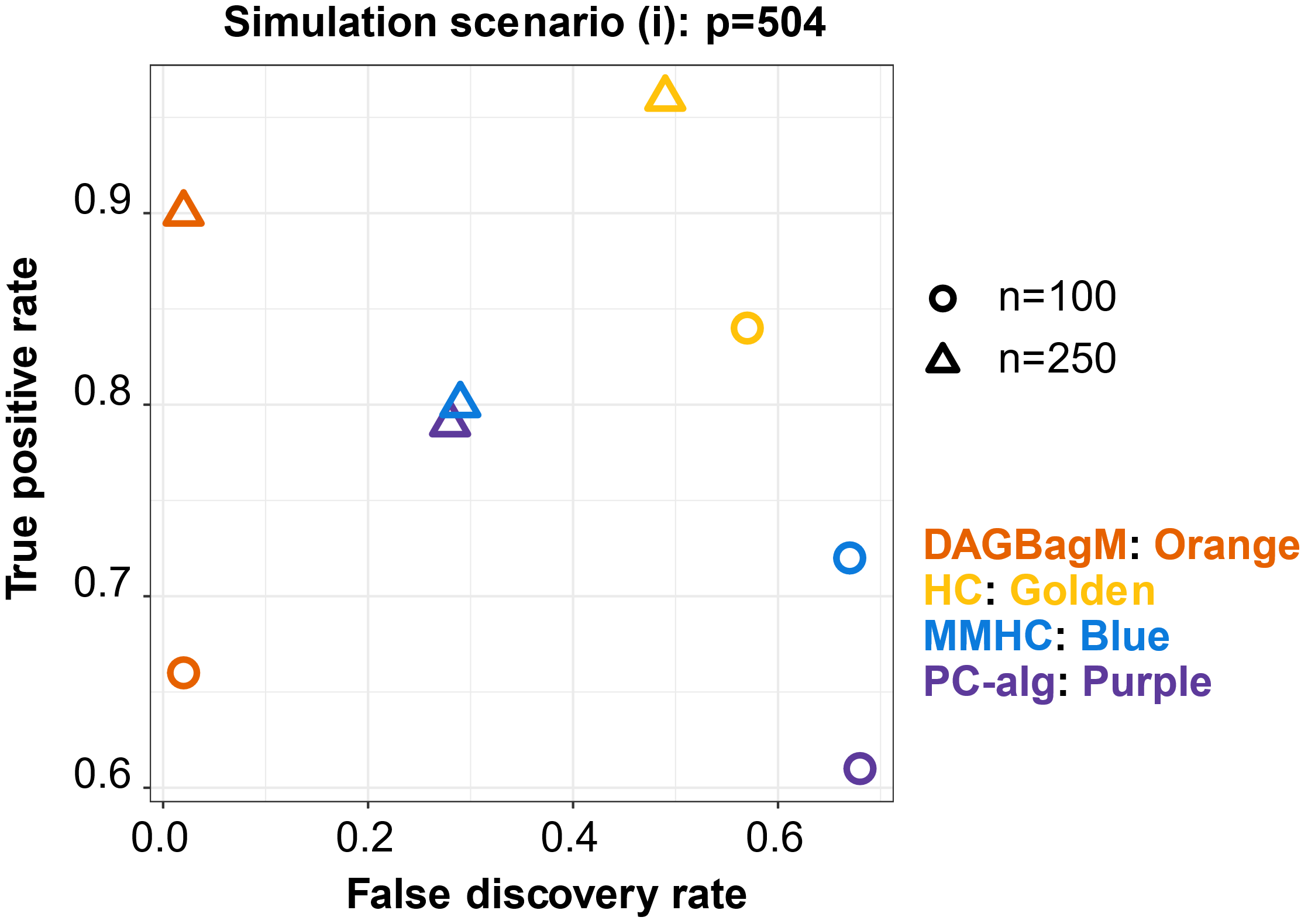


Fig. S4. Results for simulation (i) – continuous nodes. Performance in detecting the skeleton edges.

Fig. S5. Probability density curves of standard normal distribution, t-distribution with $df= 3$ and $df= 5$ and Gamma distribution with shape $= 1$, scale $= 2$.

(A)

IMMT

SAMM50

(B)

IMMT

Fig. S6. (A) shows the scatterplot of the SAMM50 protein abundance vs. its RNA expression in the Retro-ova data, and (B) shows the scatterplot for the IMMT protein abundance vs. its RNA expression in the Retro-ova data. The p-values are from the correlation test for Pearson’s product moment correlation coefficient.


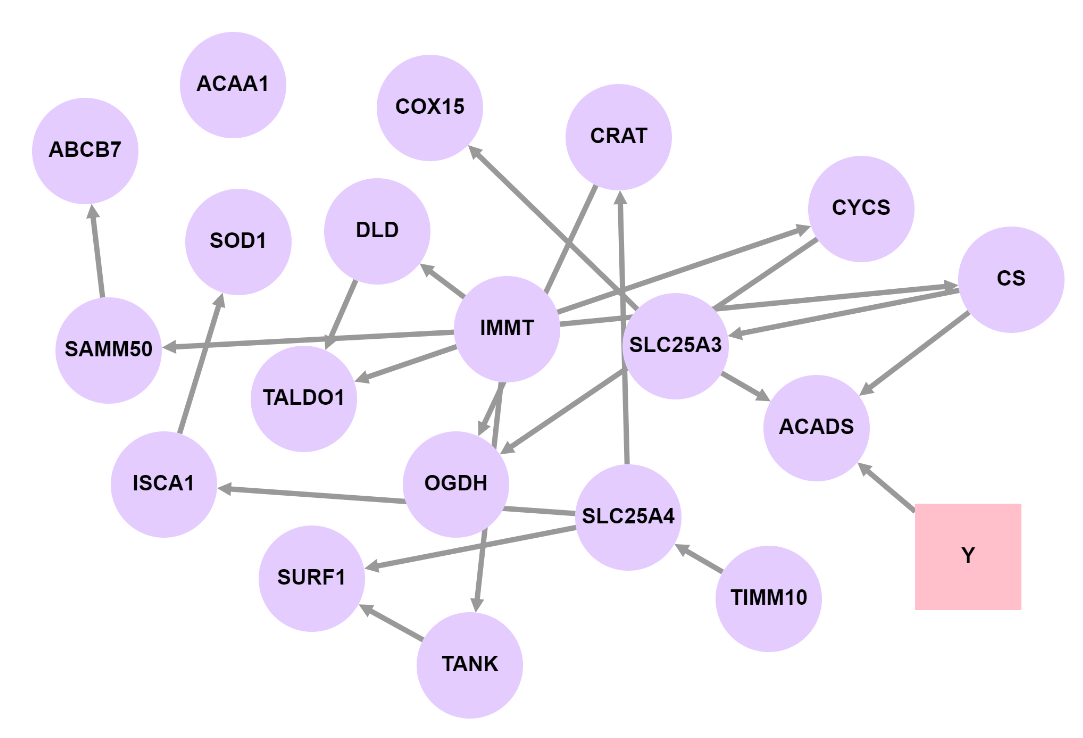


Fig. S7. DAG inferred by bnlearnD for the purple module in Fig. 6A.


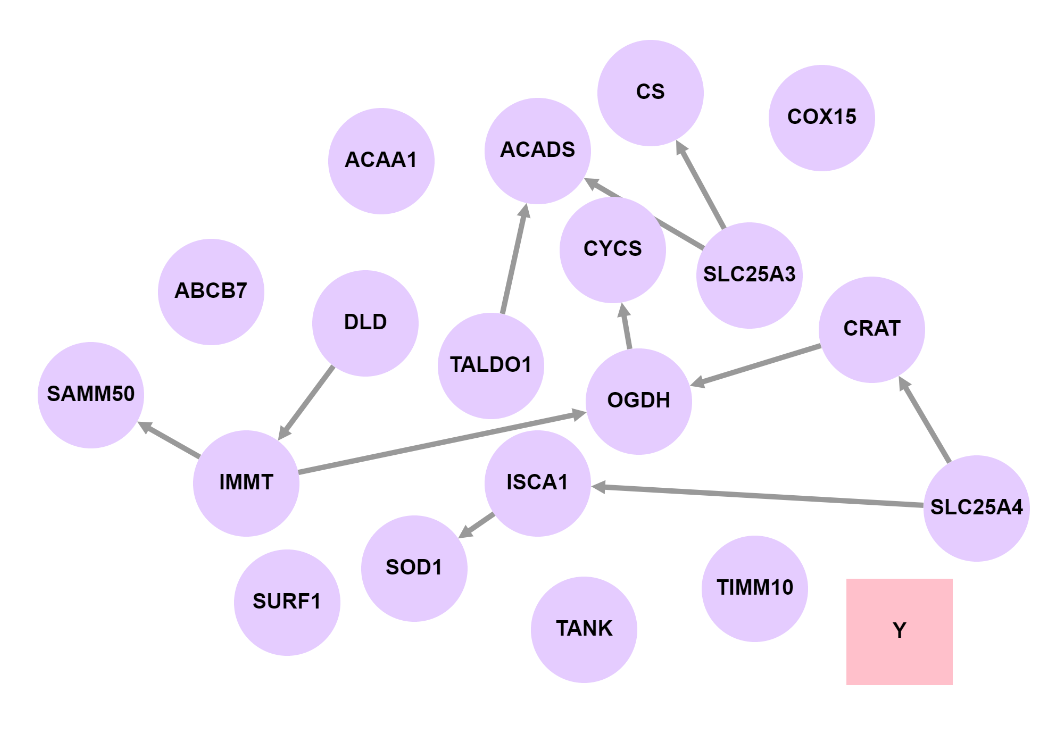


Fig. S8. DAG inferred by mDAG for the purple module in Fig. 6A.


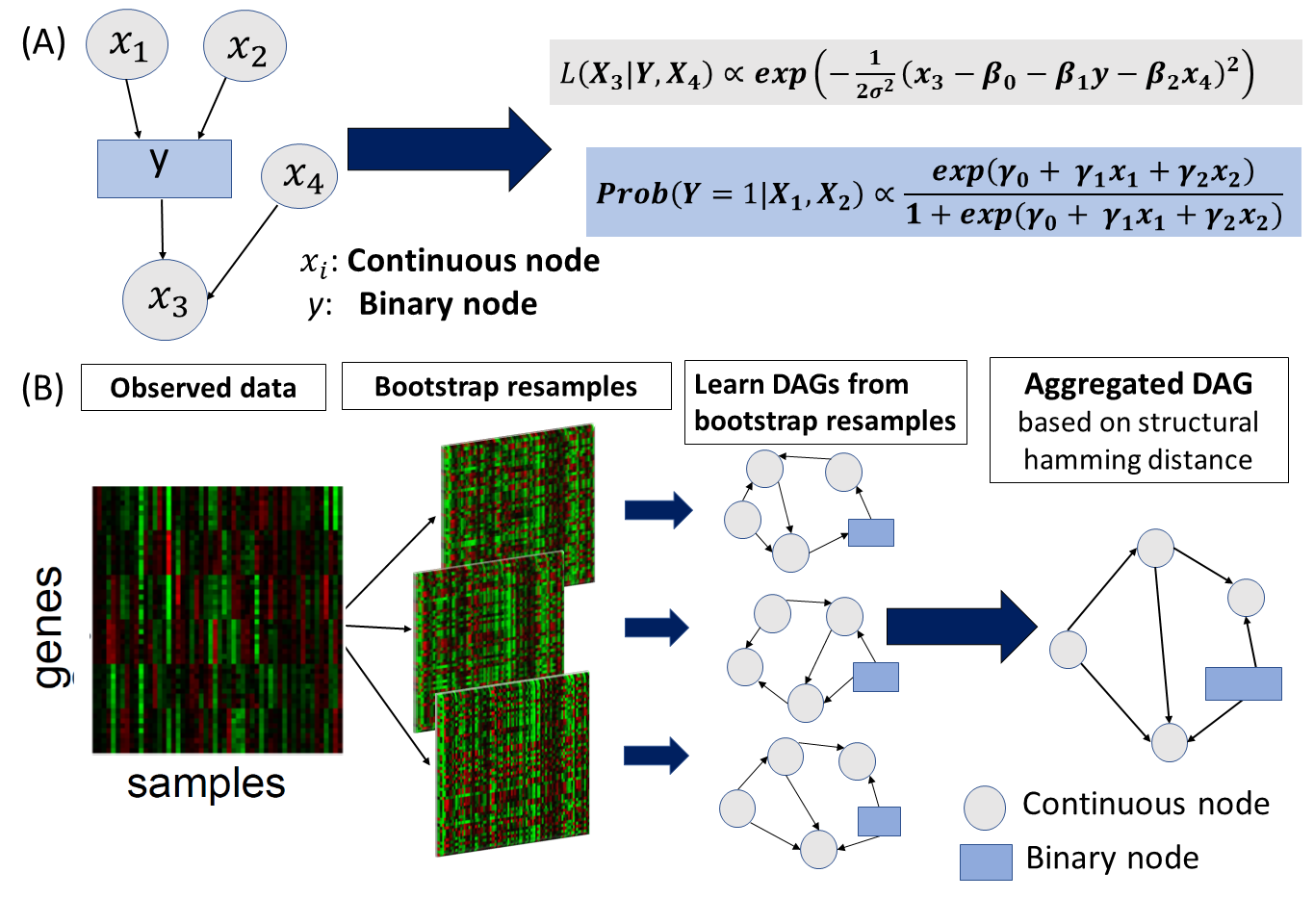


Fig. S9. DAGBagM algorithm. (A) shows a DAG with continuous nodes modeled by conditional Gaussian distributions and a binary node modeled through logistic regression. (B) shows major steps of the DAGBagM algorithm.


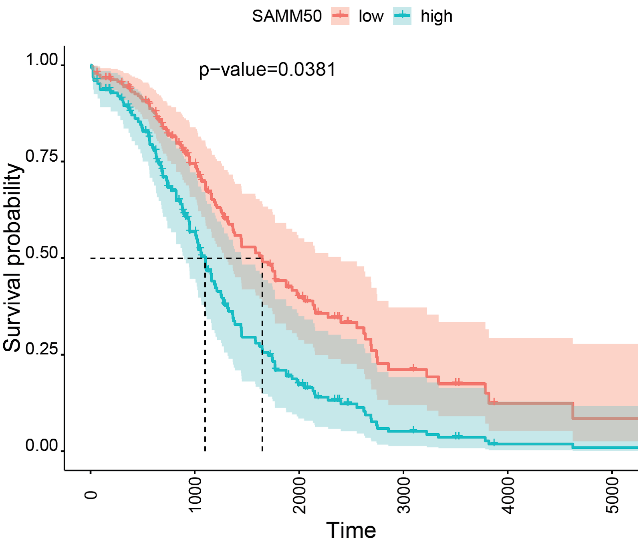

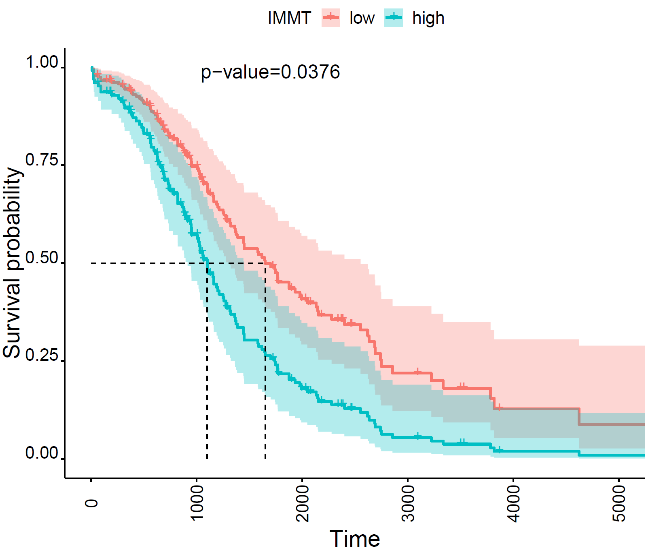


(B))

(A))

p-value = 0.0225           p-value = 0.0115

(C)


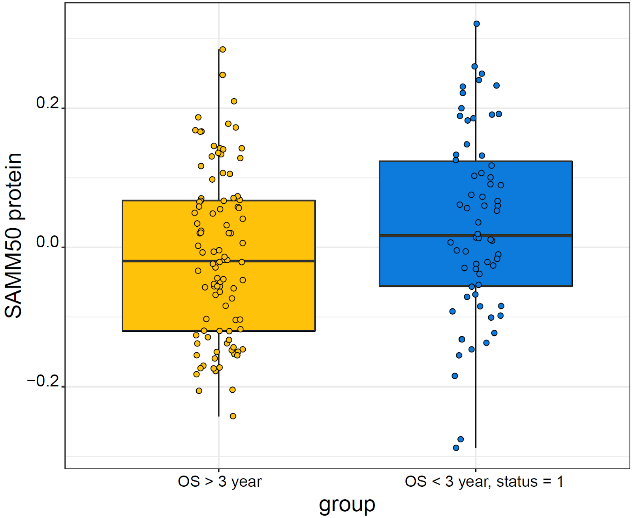

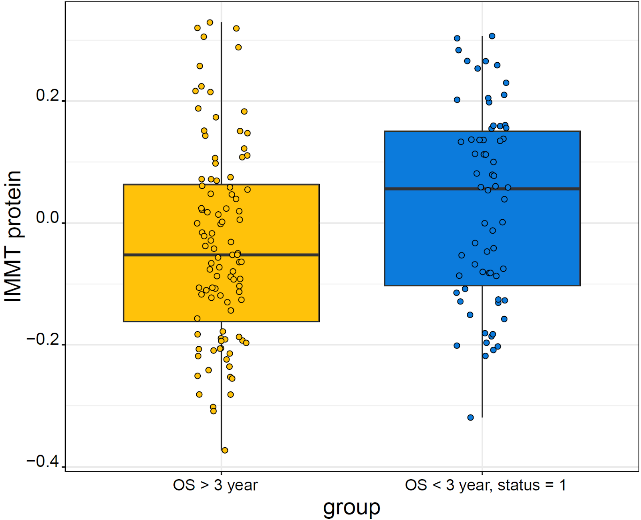


(D))

Fig S10: (A) and (B) show the estimated survival curves of the Cox models for the proteins SAMM50 and IMMT, respectively. The p-values are obtained from the Cox regression model. (C) and (D) show the distributions of protein abundances of SAMM50 and IMMT, respectively, among patients who died within 3 years and who had overall survival greater than 3 years. The p-values are from Student’s t-test.
