# Supplementary material for: DAGBagM: learning directed acyclic graphs of mixed variables with an application to identify protein biomarkers for treatment response in ovarian cancer
Source: BMC Bioinformatics. 2022 Aug 5;23:321. doi: 10.1186/s12859-022-04864-y (PMC9354326; doi:10.1186/s12859-022-04864-y)
Supplement: Supplementary file 2 — Additional file 2: Supplementary methods. [file 12859_2022_4864_MOESM2_ESM.docx]

**SUPPLEMENTARY MATERIAL**

Method S1. Efficient implementation of hill climbing algorithm

In the following, we use $\mathcal{G}$ to denote the graph in the previous step, $O^{*}$ to denote the selected operation in the previous step, and $\mathcal{G}^{*}=O^{*}(\mathcal{G})$ to denote the current graph. We also use $\delta(O:\mathcal{G})$ to denote the change of score resulting from applying operation $O$ to graph $\mathcal{G}$. We summarize the score updating and acyclic check schemes in the following two propositions [2].

**Proposition 1**: *Suppose score* $(\cdot:\cdot)$ *is a decomposable score. For an (eligible) operation* $O,\delta(O:\mathcal{G})=\delta\left( O:\mathcal{G}^{*} \right)$*, if one of the following holds:*

- $O^{*}$ *is one of the forms: "add* $x_{i}\to x_{j}$*" or "delete* $x_{i}\to x_{j}$*", and* $O$ *is not one of the forms: "add* $x_{k}\to x_{j}$*", "delete* $x_{k}\to x_{j}$*", " reverse* $x_{j}\to x_{k}$*".*
- $O^{*}$ *is of the form "reverse* $x_{i}\to x_{j}$*", and* $O$ *is not one of the forms: "add* $x_{k}\to x_{j}$*", "delete* $x_{k}\to x_{j}$*", "reverse* $x_{j}\to x_{k}$*", "add* $x_{k}\to x_{i}$*", "delete* $x_{k}\to x_{i}"$ *" "reverse* $x_{i}\to x_{k}$*".*

In short, any operation that does not involve the neighborhoods changed by the selected operation in the previous step will lead to the same score change as in the previous step. This has been pointed out by [2]. In the following, $de\left( x_{i} \right)$ denotes the set of descendants of node $x_{i}$ and $an\left( x_{i} \right)$ denotes the set of ancestors of node $x_{i}$.

**Proposition 2**: *The following holds for acyclic check.*

- *If* $O^{*}$ *is of the form "add* $x_{i^{*}}\to x_{j^{*}}$ *", then for an operation* $O$ *the following holds:*
  - - 1. *1. If* $O$ *does not lead to cycles in the previous step, then*
- *if* $O$ *is of the form "add* $x_{i}\to x_{j}$ *", and* $i\in de\left( x_{j^{*}} \right)$ *and* $j\in an\left( x_{i^{*}} \right)$*, then O leads to a cycle.*
- *if* $O$ *is of the form "reverse* $x_{i}\to x_{j}$ *", and* $j\in de\left( x_{j^{*}} \right)$ *and* $i\in an\left( x_{i^{*}} \right)$*, then O leads to a cycle.*
- *if otherwise,* $O$ *remains acyclic.*
  - 1. *2. If O leads to a cycle in the previous step, it remains cyclic.*
- *If* $O^{*}$ *is of the form "delete* $x_{i^{*}}\to x_{j^{*}}$ *", then for an operation* $O$ *the following holds:*

1. *If* $O$ *does not lead to cycles in the previous step, it remains acyclic.*
2. *If* $O$ *leads to a cycle in the previous step, then*

- *if* $O$ *is of the form "add* $x_{i}\to x_{j}$ *", and* $i\in de\left( x_{j^{*}} \right)$ *and* $j\in an\left( x_{i^{*}} \right)$*, then we need to check its acyclicity;*
- *if* $O$ *is of the form "reverse* $x_{i}\to x_{j}$ *", and* $j\in de\left( x_{j^{*}} \right)$ *and* $i\in an\left( x_{i^{*}} \right)$*, then we need to check its acyclicity;*
- *if otherwise,* $O$ *remains cyclic.*
- *If* $O^{*}$ *is of the form "reverse* $x_{i^{*}}\to x_{j^{*}}$ *", then for an operation* $O$ *the following holds:*

1. *If* $O$ *does not lead to cycles in the previous step, then*
   - *if* $O$ *is of the form "add* $x_{i}\to x_{j}$ *", and* $i\in de\left( x_{i^{*}} \right)$ *and* $j\in$ *an* $\left( x_{j^{*}} \right)$*, then O leads to a cycle.*

- *if* $O$ *is of the form "reverse* $x_{i}\to x_{j}$ *", and* $j\in de\left( x_{i^{*}} \right)$ *and* $i\in$ *an* $\left( x_{j^{*}} \right)$*, then* $O$ *leads to a cycle.*
- *if otherwise,* $O$ *remains acyclic.*

1. *If* $O$ *leads to a cycle in the previous step, then*

- *if* $O$ *is of the form "add* $x_{i}\to x_{j}$ *", and* $i\in de\left( x_{j^{*}} \right)$ *and* $j\in$ *an* $\left( x_{i^{*}} \right)$*, then we need to check its acyclicity;*
- *if* $O$ *is of the form "reverse* $x_{i}\to x_{j}$ *", and* $j\in de\left( x_{j^{*}} \right)$ *and* $i\in an\left( x_{i^{*}} \right)$*, then we need to check its acyclicity;*
- *if otherwise,* $O$ *remains cyclic.*

Method S2. Bootstrap Aggregation using hill climbing algorithm

Given an ensemble of DAGs: $\mathbb{G}^{e}=\left\{ \mathcal{G}_{b}:b=1,\cdots B \right\}$, the selection frequency (SF) of a directed edge $e$, when the reversal of an edge is counted as one unit of operation, is defined as

$$gp_{e}:=p_{e}+\frac{1}{2}p_{e^{*}}$$

where $e^{*}$ denotes the edge with the reversed direction of $e.$ $score_{d}$ can be expressed in terms of SF as given in the following proposition:

**Proposition 3**: *Given an ensemble of DAGs:* $\mathbb{G}^{e}=\left\{ \mathcal{G}_{b}:b=1,\cdots B \right\}$*, the aggregation score under* $d$ *is*

$${score}_{d}\left( \mathcal{G:}\mathbb{G}^{e} \right)=\sum_{e\in\mathbb{E(}\mathcal{G)}} \left( 1-2gp_{e} \right)+C,$$

*where*

$$C=\frac{1}{B}\sum_{b=1}^{B} \sum_{i=1}^{p} \sum_{j=1}^{p} \mathbb{A}_{b}(i,j)=\sum_{i=1}^{p} \sum_{j=1}^{p} p_{x_{i}\to x_{j}},$$

*is a constant which only depends on the ensemble* $\mathbb{G}^{e}$*, but does not depend on* $\mathcal{G}$*.*

**Proof of Proposition 3**: To help with the proof, we use Table A.1 to show the value of $S_{ij}(\mathbb{A},\mathbb{A})=max\{|\mathbb{A}(i,j)-\mathbb{A}(i,j)|$, for $1\leq i<j\leq p.$ For an adjacency matrix $\mathbb{A}$ and a given pair $(i,j)$ with $i<j$, let $(1,0)$ denote the case where $\mathbb{A}(i,j)=$ $1,\mathbb{A}(j,i)=0,(0,1)$ denote the case where $\mathbb{A}(i,j)=0,\mathbb{A}(j,i)=1$ and $(0,0)$ denote the case where $\mathbb{A}(i,j)=0,\mathbb{A}(j,i)=0$ (note $(1,1)$ is not possible due to the acyclic constraint).

Table: $S_{ij}(\mathbb{A},\mathbb{A})$ for $1\leq i<j\leq p$.

| $\mathbb{A}\setminus\mathbb{A}$ | $(1,0)$ | $(0,1)$ | $(0,0)$ |
| --- | --- | --- | --- |
| $(1,0)$ | 0 | 1 | 1 |
| $(0,1)$ | 1 | 0 | 1 |
| $(0,0)$ | 1 | 1 | 0 |

By definition:

$$\begin{matrix} {score}_{d}\left( \mathcal{G}:\mathbb{G}^{e} \right) & =\frac{1}{B}\sum_{b=1}^{B} \sum_{1\leq i<j\leq p} S_{ij}\left( \mathbb{A},\mathbb{A}_{b} \right) \\ & =\sum_{i<j:x_{i}\to x_{j}\in\mathbb{E}(\mathcal{G})} \left( p_{ji}+p_{ij}^{0} \right) \\ & +\sum_{i<j:x_{j}\to x_{i}\in\mathbb{E}(\mathcal{G})} \left( p_{ij}+p_{ij}^{0} \right) \\ & +\sum_{i<j:x_{i}\to x_{j}\notin\mathbb{E}(\mathcal{G}),x_{j}\to x_{i}\notin\mathbb{E}(\mathcal{G})} \left( p_{ij}+p_{ji} \right) , \end{matrix}$$

where $p_{ij}$ denotes the selection frequency of edge $x_{i}\to x_{j}$, and $p_{ij}^{0}=p_{ji}^{0}=1-p_{ij}-p_{ji}$ is the frequency that neither $x_{i}\to x_{j}$ nor $x_{j}\to x_{i}$ got selected.

Note that,

$$\begin{matrix} \sum_{i<j:x_{i}\to x_{j}\notin\mathbb{E}(\mathcal{G}),x_{j}\to x_{i}\notin\mathbb{E}(\mathcal{G})} \left( p_{ij}+p_{ji} \right) & =\sum_{1\leq i<j\leq p} \left( p_{ij}+p_{ji} \right) \\ & -\sum_{i<j:x_{i}\to x_{j}\in\mathbb{E}(\mathcal{G})} \left( p_{ij}+p_{ji} \right)-\sum_{i<j:x_{j}\to x_{i}\in\mathbb{E}(\mathcal{G})} \left( p_{ij}+p_{ji} \right). \end{matrix}$$

Therefore

$$\begin{matrix} {score}_{d}\left( \mathcal{G}:\mathbb{G}^{e} \right) & =\sum_{i<j:x_{i}\to x_{j}\in\mathbb{E}(\mathcal{G})} \left( p_{ji}+1-2\left( p_{ij}+p_{ji} \right) \right) \\ & +\sum_{i<j:x_{j}\to x_{i}\in\mathbb{E}(\mathcal{G})} \left( p_{ij}+1-2\left( p_{ij}+p_{ji} \right) \right) \\ & +\sum_{1\leq i<j\leq p} \left( p_{ij}+p_{ji} \right) \\ & =\sum_{i<j:x_{i}\to x_{j}\in\mathbb{E}(\mathcal{G})} \left( 1-2\left( p_{ij}+\frac{1}{2}p_{ji} \right) \right) \\ & +\sum_{i<j:x_{j}\to x_{i}\in\mathbb{E}(\mathcal{G})} \left( 1-2\left( p_{ji}+\frac{1}{2}p_{ij} \right) \right) \\ & +\sum_{1\leq i<j\leq p} \left( p_{ij}+p_{ji} \right). \end{matrix}$$

By definitions of the constant $C$ and the selection frequency, we complete the proof.

Because of Proposition 3, the hill climbing search algorithm with ${score}_{d}$ can be simplified to the procedure described in Algorithm 1.

Algorithm S1: Hill climbing algorithm for DAG aggregation

**Input**: an ensemble of DAGs: $\mathbb{G}^{e}=\left\{ \mathcal{G}_{b}:b=1,\cdots B \right\}$.

Calculate selection frequency (SF) $gp_{e}$ for all possible edges.

Order edges with SF > 50%.

Add edges sequentially according to $\mathrm{SF}$ and stop when $SF\leq0.5$.

- Initial step: $\mathcal{G}^{(0)}=$ empty graph
- $s^{th}$ step: current graph $\mathcal{G}^{(s)}$, current operation $O$ : "add the edge with the $s^{th}$ largest SF".
- If $O$ passes the acyclic check, then $\mathcal{G}^{(s+1)}=O\left( \mathcal{G}^{(s)} \right)$, i.e., add this edge.

If $O$ does not pass the acyclic check, then $\mathcal{G}^{(s+1)}=\mathcal{G}^{(s)}$, i.e., does not add this edge

- If the $(s+1)^{th}$ largest $SF>50\%$, proceed to step $s+1$.

Otherwise, set $\mathcal{G}^{*}=\mathcal{G}^{(s+1)}$ and stop the algorithm.

**Output**: $\mathcal{G}^{*}$

Method S3. Integrative DAG learning pipeline

| Integrative DAG learning pipeline for real data application |
| --- |
| 1: Denote $D_{1}$ as the pre-processed cell line proteomics data consisting of $n_{1}=18$ samples from $t_{1}=8hr$ post-treatment and another $n_{2}=18$ samples from $t_{2}=24hr$ post-treatment. Denote $D_{2}$ as the pre-processed Retro-ova data.  2: $M_{G}=\left\{ g_{i} \right\}_{i=1}^{G}$ represents the index set of the $G=260$ proteins from Adipogenesis and Oxidative Phosphorylation pathways that were observed in both $D_{1}$ and $D_{2}$.  3: Denote $A_{G\times18}$ and $B_{G\times18}$ as the protein intensity matrix of proteins in $M_{G}$ at $t_{1}$ and $t_{2}$ respectively in the cell line data set. Stacking A and B together results $C_{(8+24)}$, which is a matrix of dimension $2G\times n_{1}$.  4: Applying DAGBagM to $C_{(8+24)}$ with blacklist $BL$ resulted in $C_{\text{est }}$, a DAG with $2G$ nodes. $BL$ is used to suppress all edges from (i) $g_{i}$ to $g_{j}$ at $t_{1}$, (ii) $g_{i}$ to $g_{j}$ at $t_{2}$, and (iii) $g_{i}$ at $t_{2}$ to $g_{j}$ at $t_{1}$, for all $g_{i},g_{j}\in M_{G}$.  5: Extract the top off-diagonal block matrix $(G\times G)$ corresponding to the estimated edges directed from proteins at $t_{1}$ to proteins at $t_{2}:$ call it $C_{est}(8\to24)$. Since there might be an edge from protein $g_{i}$ at $t_{1}$ to protein $g_{j}$ at $t_{2}$ and another edge from $g_{j}$ at $t_{1}$ to $g_{i}$ at $t_{2},C_{est}(8\to24)$ is not necessarily a DAG. In the next step, we select a subset of edges to pass as whitelist for learning a DAG from $D_{2}$.  6: Randomly sample $90\%$ edges from $C_{est}(8\to24)$ and sequentially add them to an empty graph with a check of acyclic status at every step until the check fails. Denote $C_{est(8\to24)}^{*}$ as the last DAG before the acyclic check fails. Repeat this step 100 times and denote the corresponding DAGs as $C_{b est(8\to24)}^{*}$, $b=1,\ldots, 100$.  7: Generate $B = 100$ bootstrap resamples of the data $D_{2}$: call them $D_{b}$, $b = 1, \ldots, B$.  For each $b$, learn a DAG based on $D_{b}$ with $C_{b est(8\to24)}^{*}$ as prior (passed as whitelist).  Aggregate $100$ DAGs using our proposed aggregation procedure and call it $P_{est}$.  8: Identify closely linked modules with $10\sim20$ proteins based on $P_{est}$.  9: Denote the treatment response vector of the $n_{3} = 79$ ovarian patients of the  Retro-ova data $D_{2}$ as $Y : yi = 1$ for sensitive, and $yi = 0$ for resistant/refractory.  10: For each module of $P_{est}$, apply DAGBagM to learn a DAG for proteins in the module together with $Y$employing the same procedure to use prior information from $C_{b est(8\to24)}^{*}$ as described in step 6. |

Method S4. Application: Ovarian Cancer Proteomics Data -- Pre-processing of the datasets used in two steps

We performed global normalization to align the sample median to remove any systematic variation across the samples for all the datasets. We then filtered out the proteins that are missing all samples in a given batch (batch-level missing). For the Retro-ova data we again filtered out proteins that were missing from $75\%$ of the samples. We then applied batch correction to all datasets using an $R$ tool: ComBat [1] to remove batch-effect. We also used an imputation tool DreamAI [3] to impute the Retro-ova data.

**References**

1. Johnson, W., Li, C., and Rabinovic, A. (2007). Adjusting batch effects in microarray data using empirical bayes methods. Biostatistics, $8(1):118a127$.
2. Koller, D. and Friedman, N. (2009). Probabilistic graphical models: principles and techniques. MIT press.
3. Ma, W., Kim, S., Chowdhury, S., Li, Z., Yang, M., Yoo, S., Petralia, F., Jacobsen, J., Li, J.J., Ge, X., et al. (2021). DreamAI: algorithm for the imputation of proteomics data. bioRxiv.
